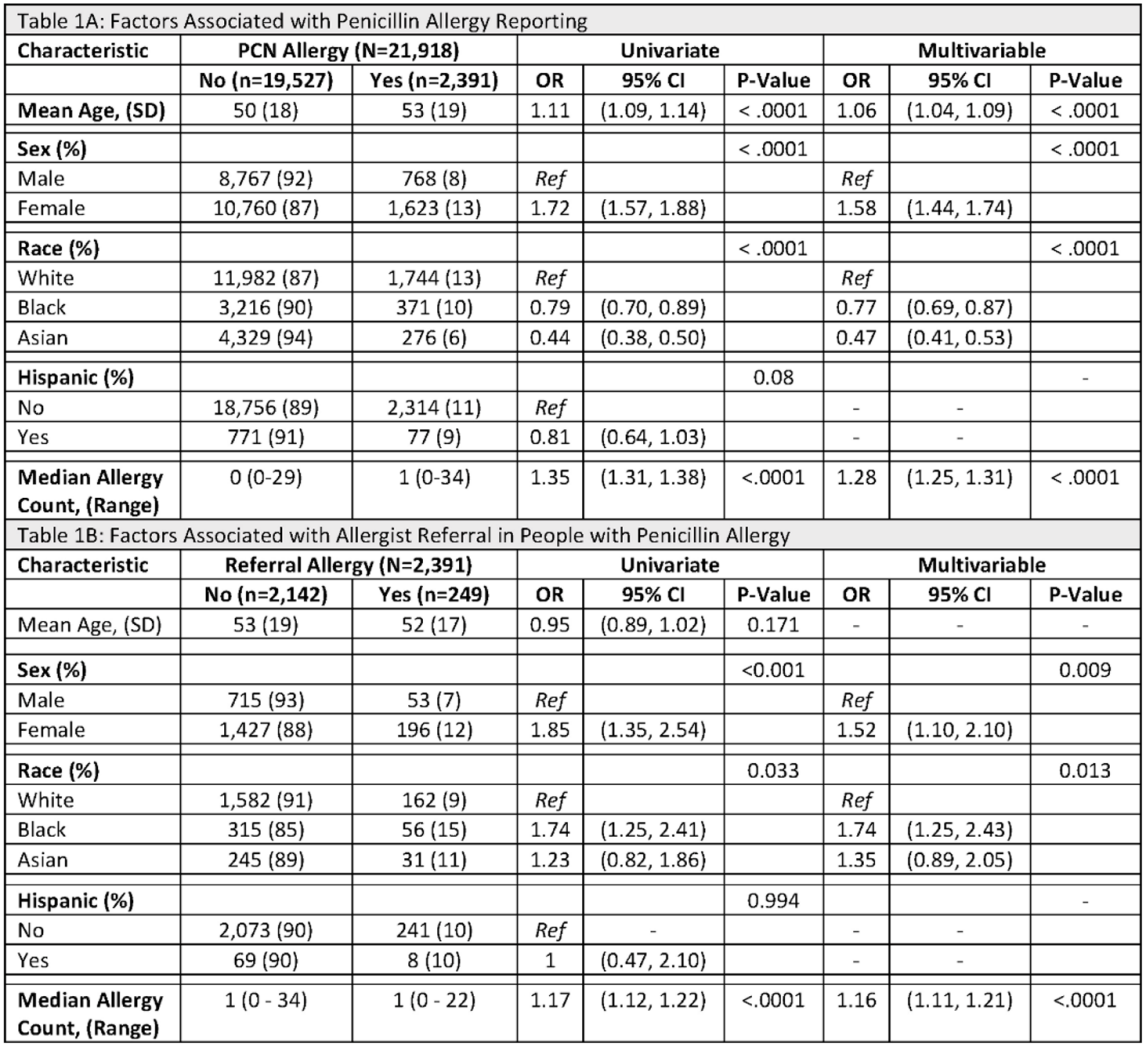# Racial and ethnic differences in penicillin allergy reporting and allergist referral

**DOI:** 10.1017/ash.2022.96

**Published:** 2022-05-16

**Authors:** Charles Bornmann, Christina Ortiz, Rubeen Guardado, Joseph Gillis, Kristin Huang, Kimberly Blumenthal, Shira Doron, Maureen Campion, Alysse Wurcel

## Abstract

**Background:** Antimicrobial resistance (AMR) is a global public health crisis. A key strategy to combat AMR is to use targeted antibiotics, which is difficult in patients who report an allergy to penicillin. Increased risk for resistant infections, mortality, and healthcare costs are associated with penicillin allergies; however, up to 90% of those with a reported penicillin allergy do not have a true allergy. We investigated racial and ethnic differences related to penicillin allergy delabeling by analyzing rates of penicillin allergy reporting and referral for allergist consultation. **Methods:** Tufts Medical Center is a teaching medical center in Boston, Massachusetts. This study cohort contains all patients seen in 2019 by clinicians at Primary Care Boston, the main primary care practice at Tufts Medical Center. Demographic data, documented allergies, and referral history were collected from the electronic medical record. We performed univariate and multivariable analyses using logistic regression models. Covariates found to be statistically significant (*P* < .05) in the univariate analyses were included in the multivariable model. **Results:** ***In total***, 2,391 (11%) patients reported a penicillin allergy, but only 249 (10%) were referred to an allergist (Table 1). Black patients and Asian patients were less likely to report a penicillin allergy than White patients. We detected no differences related to Hispanic ethnicity. Black patients with penicillin allergy were more likely to be referred to an allergist. **Conclusions:** There were low rates of allergist referral for penicillin allergy delabeling in this cohort. We identified racial differences in both penicillin allergy reporting and allergist referral. Allergist consultation is an important opportunity to combat AMR and should be considered for all patients reporting a penicillin allergy. Future work should evaluate equitable access to allergy delabeling resources.

**Funding:** None

**Disclosures:** None

**Table:**